# The Macromolecular Basis of Phytoplankton C:N:P Under Nitrogen Starvation

**DOI:** 10.3389/fmicb.2019.00763

**Published:** 2019-04-17

**Authors:** Justin D. Liefer, Aneri Garg, Matthew H. Fyfe, Andrew J. Irwin, Ina Benner, Christopher M. Brown, Michael J. Follows, Anne Willem Omta, Zoe V. Finkel

**Affiliations:** ^1^Department of Geography and Environment, Mount Allison University, Sackville, NB, Canada; ^2^Department of Mathematics and Computer Science, Mount Allison University, Sackville, NB, Canada; ^3^Department of Earth, Atmospheric and Planetary Science, Massachusetts Institute of Technology, Cambridge, MA, United States

**Keywords:** phytoplankton, diatoms, prasinophytes, stoichiometry, nitrogen, macromolecules

## Abstract

Biogeochemical cycles in the ocean are strongly affected by the elemental stoichiometry (C:N:P) of phytoplankton, which largely reflects their macromolecular content. A greater understanding of how this macromolecular content varies among phytoplankton taxa and with resource limitation may strengthen physiological and biogeochemical modeling efforts. We determined the macromolecular basis (protein, carbohydrate, lipid, nucleic acids, pigments) of C:N:P in diatoms and prasinophytes, two globally important phytoplankton taxa, in response to N starvation. Despite their differing cell sizes and evolutionary histories, the relative decline in protein during N starvation was similar in all four species studied and largely determined variations in N content. The accumulation of carbohydrate and lipid dominated the increase in C content and C:N in all species during N starvation, but these processes differed greatly between diatoms and prasinophytes. Diatoms displayed far greater accumulation of carbohydrate with N starvation, possibly due to their greater cell size and storage capacity, resulting in larger increases in C content and C:N. In contrast, the prasinophytes had smaller increases in C and C:N that were largely driven by lipid accumulation. Variation in C:P and N:P was species-specific and mainly determined by residual P pools, which likely represent intracellular storage of inorganic P and accounted for the majority of cellular P in all species throughout N starvation. Our findings indicate that carbohydrate and lipid accumulation may play a key role in determining the environmental and taxonomic variability in phytoplankton C:N. This quantitative assessment of macromolecular and elemental content spanning several marine phytoplankton species can be used to develop physiological models for ecological and biogeochemical applications.

## Introduction

**FIGURE 1:**
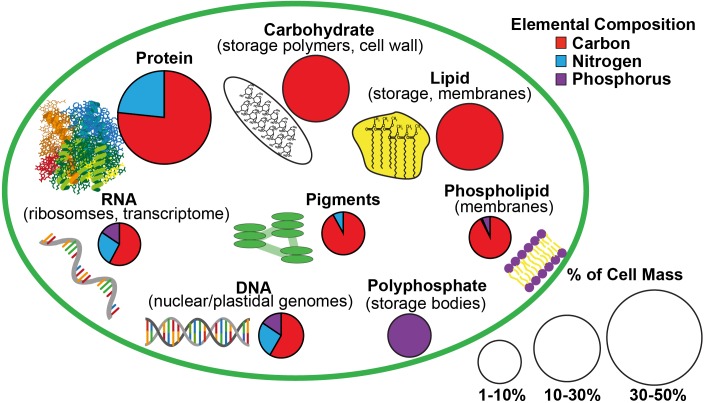
A schematic of the estimated macromolecular content of a microalgal cell and the allocation of carbon, nitrogen, and phosphorus to each macromolecular pool based on Geider and LaRoche (2002) and Dyhrman (2016). The size of the pie charts indicate the estimated contribution of each macromolecular pool to total cell mass while the portions of the pie charts indicate the relative content of carbon, nitrogen, and phosphorus in each type of macromolecule.

The elemental stoichiometry (C:N:P) of phytoplankton and particulate organic matter (POM) in the surface ocean is often assumed to conform to the Redfield ratio of 106:16:1 (Redfield, 1958). However, the C:N:P of phytoplankton and of surface POM, largely derived from phytoplankton and their detritus, can greatly deviate from Redfield proportions (Quigg et al., 2003; Martiny et al., 2013a,b; Garcia et al., 2018). This variability in phytoplankton C:N:P may influence key biogeochemical cycles (Finkel et al., 2010) by affecting how efficiently phytoplankton biomass is remineralized by bacteria (Del Giorgio and Cole, 1998), exported to the deep ocean (Weber and Deutsch, 2010), or utilized by consumers (Sterner and Elser, 2002; Hessen et al., 2004). The C:N:P of phytoplankton reflects their macromolecular content (e.g., protein, carbohydrate, lipid, and nucleic acids; summarized in Figure 1; Elser et al., 2000; Geider and LaRoche, 2002; Finkel et al., 2016), which varies among major phytoplankton groups (Finkel et al., 2016) and within a species in response to environmental conditions (Geider and LaRoche, 2002). This phylogenetic and physiological variability in phytoplankton macromolecules may provide a mechanistic basis for modeling phytoplankton and ocean particulate C:N:P (Geider and LaRoche, 2002; Follows and Dutkiewicz, 2011).

Fixed nitrogen is the limiting factor for phytoplankton growth over much of the global ocean (Moore et al., 2013) and its availability affects the C:N:P of phytoplankton cells (Geider and LaRoche, 2002; Hillebrand et al., 2013). When N limits phytoplankton growth, C:N tends to increase and N:P tends to decline (Rhee, 1978; Goldman et al., 1979; Elrifi and Turpin, 1985; Leonardos and Geider, 2004; Garcia et al., 2016). The macromolecular basis of this response is a decline in cell protein content and a reallocation of remaining protein content from photosynthetic and biosynthetic (e.g., ribosomes) components to N metabolism and acquisition (Young and Beardall, 2003; Hockin et al., 2012; Simionato et al., 2013). Nitrogen stress also tends to result in the accumulation of C and energy as carbohydrate or lipid (Piorreck et al., 1984; Breuer et al., 2012; Zhu et al., 2014). Many culture studies of phytoplankton C:N:P have examined responses to balanced, steady-state N limitation, but nutrient stress also occurs as unbalanced nutrient starvation and arrested growth due to rapid nutrient fluctuations in dynamic ocean environments (Goldman, 1988; Legendre and Rassoulzadegan, 1995). Unbalanced N starvation may also have a distinct effect on phytoplankton C:N:P as it has been shown to cause larger accumulations of C-rich lipid (Richardson et al., 1969; Lacour et al., 2012b) and larger decreases in N-rich proteins (Geider et al., 1993; Berges et al., 1996; Geider et al., 1998) compared to steady-state N limitation (Cullen et al., 1992; Halsey et al., 2013).

Storage macromolecules may play a particularly large role in the variability of phytoplankton C:N:P as they can represent large fractions of these elements and their utilization differs among phytoplankton taxa (Allen, 1984; Geider and LaRoche, 2002; Gillooly et al., 2005). The accumulation of C-rich carbohydrate and lipid storage during N stress varies among phytoplankton taxa, both in extent and in the relative distribution between these two pools (Piorreck et al., 1984; Gatenby et al., 2003; Breuer et al., 2012). A preference for carbohydrate or lipid storage may affect a species’ growth strategy as these storage products differ in their energetic efficiency (Sorguven and Ozilgen, 2013), utilization of intracellular space (Subramanian et al., 2013), and effect on cell buoyancy (Raven, 1984; Richardson and Cullen, 1995). Additionally, the few comprehensive studies of phytoplankton P allocation show the majority of total P is not accounted for by P-rich functional macromolecules (RNA, DNA, and phospholipids), particularly under N-limited conditions (Rhee, 1978; Leonardos and Geider, 2004; Mouginot et al., 2015; Garcia et al., 2016). The large amount of residual P not detected in these functional macromolecules is typically assumed to be in storage pools like polyphosphate bodies and orthophosphate in vacuoles that are difficult to quantify (Miyata et al., 1986; Diaz and Ingall, 2010; Martin et al., 2014; Dyhrman, 2016). The effect of C and P storage on phytoplankton C:N:P may vary with cell size as storage capacity may be greater in larger cells (Grover, 1991; Tozzi et al., 2004; Gillooly et al., 2005) and a greater benefit to larger cells in dynamic light and nutrient environments (Talmy et al., 2014).

Using macromolecular variation as a mechanistic basis to explain ocean C:N:P variability requires a comprehensive understanding of how elemental allocation to macromolecules differs among phytoplankton taxa and ocean conditions. Here we examine the effect of N starvation on phytoplankton elemental and macromolecular content accounting for all major C- and N-containing pools (protein, carbohydrate, lipid, pigments). We measured the major functional P-containing pools (RNA, DNA, phospholipids) and total P and describe the P not attributed to measured functional pools as residual P, which may be intracellular storage. Cell size and evolutionary history likely affect macromolecular storage capacity and adaptation to different nutrient regimes may affect the overall plasticity of macromolecular content across species. Hence we examined two phytoplankton classes, diatoms and prasinophytes, which represent distinct size classes and adaptation to different environmental niches. Diatoms are typically opportunists that exploit dynamic nutrient conditions (Sarthou et al., 2005) while the smaller prasinophytes appear to be adapted to more stable nutrient conditions (Cardol et al., 2008; Six et al., 2009). Additionally, C:N appears to be lower in prasinophytes than diatoms during nutrient replete conditions (Quigg et al., 2003; Garcia et al., 2018), possibly due to a relatively higher protein content in prasinophytes (Finkel et al., 2016). Portions of the macromolecular response to steady-state N limitation in diatoms and prasinophytes have been well characterized (Leonardos and Geider, 2004; Halsey et al., 2014; Halsey and Jones, 2015), yet it is unclear how the macromolecular response of these groups to non-steady state N starvation may differ. We anticipated that diatoms would display greater C:N:P variability as their larger cell size may allow more storage accumulation of carbohydrates and lipids and their adaptation to dynamic nutrient regimes may allow more variation in their N-rich functional macromolecules (protein, RNA, and pigments). We also hypothesized that protein, the largest fraction of phytoplankton N, would be the most variable macromolecular pool during N starvation and dominate variation in C:N and N:P. We find dramatic differences between diatoms and prasinophytes with regard to the impact of N starvation on C:N:P ratios, largely due to varying utilization of C-rich storage macromolecules and species-specific differences in P pools likely associated with storage.

## Materials and Methods

### Study Species and Growth Conditions

The diatoms *Thalassiosira pseudonana* (strain CCMP 1335) and *Thalassiosira weissflogii* (strain CCMP 1010) as well as the Arctic prasinophyte *Micromonas* sp. (strain CCMP 2099) were obtained from the National Center for Marine Algae and Microbiota (NCMA). The prasinophyte *Ostreococcus tauri* (strain OTH95, RCC745) was obtained from the Roscoff Culture Collection (RCC). The strains of *O. tauri* (mean cell volume of 1.8 ± 0.3 *μ*m^3^) and *T. pseudonana* (mean cell volume of 158 ± 23 μm^3^) used are both coastal isolates while the strains of *Micromonas* sp. (mean cell volume of 1.8 ± 0.3 μm^3^) and *T. weissflogii* (mean cell volume of 1630 ± 215 μm^3^) were isolated from shelf waters of the Arctic Ocean and the Gulf Stream of the North Atlantic, respectively. Cell volumes for each strain were based on measurements of a minimum of 50 live, unstained nutrient-replete cells observed by light microscopy and assuming a cylindrical form for diatoms and a spherical form for both prasinophytes (Hillebrand et al., 1999).

All cultures were grown under a irradiance of 85 μmol photons m^-2^ s^-1^ provided by cool white fluorescent bulbs on a 12:12 light:dark cycle and a temperature of 18°C with the exception of *Micromonas* sp., which was grown at 6°C. These conditions have been shown to be at subsaturating growth irradiance and near optimum growth temperatures for each study species (Thompson, 1999; Strzepek and Price, 2000; Lovejoy et al., 2007; Six et al., 2008). Growth media was prepared from natural seawater (Cape Tormentine, Canada) with a salinity of ∼32 ppt and amended with half the f/2 concentrations (Guillard and Ryther, 1962; Guillard, 1975) of sodium phosphate, sodium silicate (not used for prasinophytes), trace metals, and vitamins. Media was further amended with 2 mM sodium bicarbonate and 60 or 120 μM sodium nitrate, respectively, for diatoms and prasinophytes. The lower nitrogen (N) concentration was necessary for diatom cultures to ensure carbon-replete conditions and a maximum pH of less than 9 at all growth phases. Media was adjusted to a pH of 7.95–8.00 with HCl and filter sterilized (Pall Acropak 0.8/0.2 μm capsule filter) before use. Cultures were maintained in 5 L glass bottles (Pyrex) and mixed by stirring with PTFE stir bars at ∼60 RPM and continuous bubbling with filter-sterilized (VWR, 0.2 μm PES syringe filter) air. To assess cell composition during nutrient-replete balanced exponential growth, all cultures were maintained as optically thin, semi-continuous batch cultures and were considered to be fully acclimated to these conditions after a minimum of 10 generations with less than 15% variation in growth rate. During this acclimation period cultures were maintained within ∼15–120% of the cell density at the time of sampling for cell composition to ensure similar optical conditions and growth rates throughout this period. After each culture was sampled at fully acclimated, nutrient-replete exponential growth, N starvation was imposed by diluting these cultures once with N-free media (the same media as described above without sodium nitrate added). This single dilution with N-free media was such that cultures reached a similar optical density and pH in stationary phase as observed during the nutrient-replete exponential phase.

### Sampling

**FIGURE 2:**
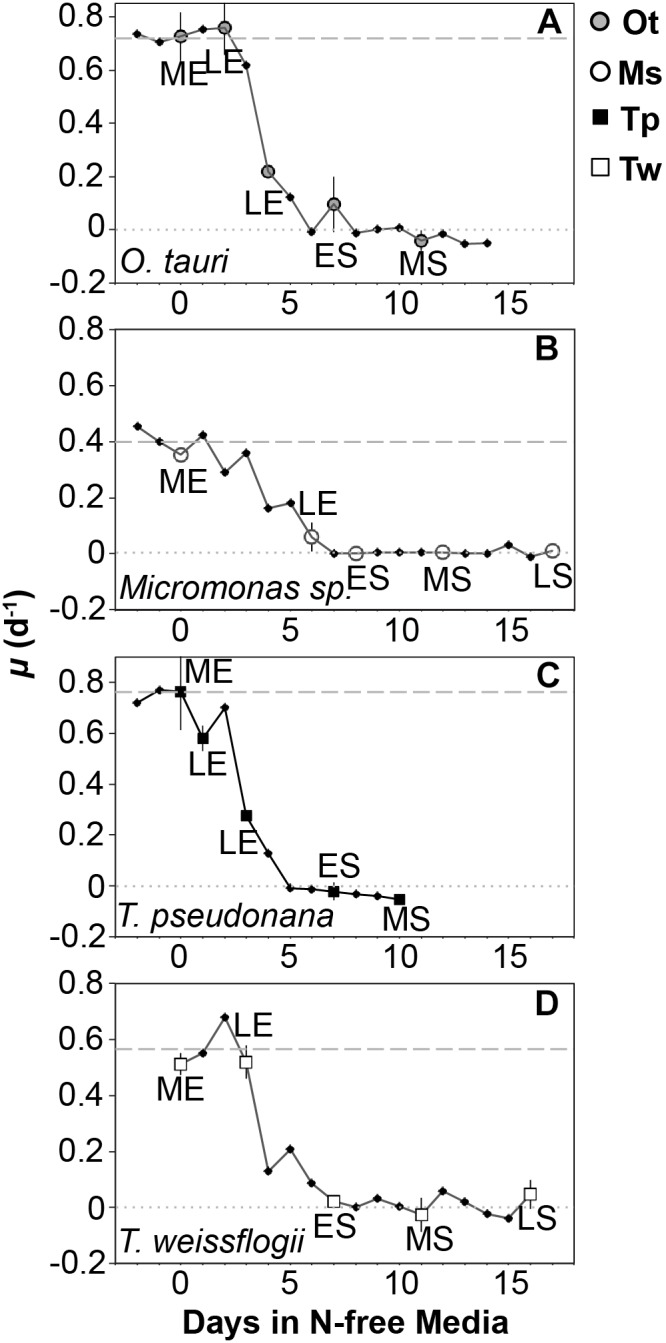
Growth rate (μ, d^-1^) from N-replete balanced growth to N starvation in **(A)** *T. pseudonana*, **(B)** *T. weissflogii*, **(C)** *O. tauri*, and **(D)** *Micromonas* sp. The larger symbols also shown in the legend indicate sampling points for macromolecular composition at mid-exponential (ME), late-exponential (LE), early-stationary (ES), mid-stationary (MS), and late-stationary (LS) growth phases. Criteria for defining these sampling points are provided in the text. The dashed line indicates the *μ_max_* for a species determined over a minimum of 10 generations during N-replete, balanced growth. The dotted line indicates a growth rate of 0. Error bars indicate one standard deviation among triplicate cultures.

Triplicate cultures were sampled daily for cell density by collecting and preserving a 1–3 ml aliquot of each culture replicate. *T. pseudonana* and *Micromonas* sp. were preserved in 0.5% glutaraldehyde and *T. weissflogii* and *O. tauri* were preserved in 2% Lugol’s solution. The preservation technique used for each study species was based on preliminary tests of various concentrations of Lugol’s solution and glutaraldehyde (0.5, 1, and 2%) to select the method that resulted in no significant change compared to the initial cell density of unpreserved cultures. Cell density in all samples was determined within 2 h of collection by light microscopy using a hemocytometer except for samples of *T. weissflogii*, which were counted using a Sedgwick-Rafter chamber. Cell density was used to track the growth rate of each culture with the onset of N starvation to determine sampling points for cell composition (Figure 2). Growth rate (μ) was calculated as the daily exponential rate of change in cell density: μ (d^-1^) = ln(*N*_f_ /*N*_i_ )/*dt*, where *N*_f_ is the cell density at a given sampling point, *N*_i_ is the cell density from the previous day measured at the same time of day and *dt* is the time interval in days between these two sampling points.

Samples for elemental and macromolecular composition, dissolved nutrient concentrations, and bacterial contamination were collected during nutrient-replete, balanced exponential growth and at four additional points spanning late-exponential and stationary phase (Figure 1). Late-exponential sampling points characterized cell composition during the onset of N starvation 1–5 days after dilution to N-free media in each experiment. Stationary phase sampling was performed at early-stationary phase near N-starved cessation of growth (6–7 days after dilution to N-free media), at mid-stationary phase 6 days after the cessation of growth (10–12 days after dilution to N-free media) and at late-stationary phase either 10 days after cessation of growth (*T. weissflogii* and *Micromonas* sp.) or when cell densities consistently declined for 3 days after the mid-stationary phase (*T. pseudonana* and *O. tauri*) depending on whichever event occurred first. Element and macromolecular data from the late-stationary sampling of *T. pseudonana* and *O. tauri* are not presented or included in our data analyses as bacterial biomass was estimated to be greater than 10% of phytoplankton biomass at these sampling points (see Bacterial Enumeration section for details). Two additional samples were also collected for elemental composition only in late-exponential phase and stationary phase.

Samples for cell composition were collected by filtration under gentle vacuum pressure (<18 kPa or 5 in Hg) and low light. Samples for particulate carbon (C), nitrogen (N), phosphorus (P), carbohydrate, lipid, and pigments were collected on pre-combusted (4 h at 450°C) Whatman GF/F filters (effective pore size, 0.7 μm). Samples for protein, RNA, and DNA were collected on Whatman Nucleopore 25 mm polycarbonate membrane filters. Different filter types were used across these samples due to the varying compatibility of filter materials with a particular macromolecular assay. Polycarbonate filters with a pore size of 0.8 μm were used for both diatom species, while filters with a pore size of 0.4 and 0.6 μm were used for *O. tauri* and *Micromonas* sp., respectively, as these pore sizes were shown to be required to fully retain these smaller species. Filtrate from samples collected on pre-combusted GF/F filters was collected for dissolved nutrient analyses. All macromolecule samples were frozen immediately after collection in liquid nitrogen and stored at -80°C. Samples for particulate C, N, P and dissolved nutrient analyses were immediately placed in a -20°C freezer after collection. Carbohydrate, lipid, and pigment samples were freeze-dried prior to analyses to prevent dilution of the extracting solutions used in each analysis by variable amounts of seawater (∼100–200 μl) retained on GF/F filters. Abundance of contaminating bacteria was sampled by preserving 1 ml aliquots of each culture replicate in 0.1% glutaraldehyde (electron microscopy-rade, Sigma #G5882). Bacterial samples were allowed to fix at room temperature for 15 min, then frozen in liquid nitrogen and stored at -80°C.

### Elemental and Nutrient Analyses

Filters collected for particulate C and N were dried at 60°C for 2 days, pelleted in pressed tin capsules and analyzed with a Costech CHN analyzer using acetanilide as a standard.

Samples for total particulate phosphorus were dried and extracted by hydrolysis with 0.1M HCl at 90°C (Solorzano and Sharp, 1980). Phosphorus was quantified by the ammonium molybdate method (Chen et al., 1956), modified for a microplate format, using a SpectraMax M3 microplate reader (Molecular Devices). Collected filtrate was thawed immediately before analyses and dissolved inorganic nitrogen (nitrate, nitrite, and ammonium), phosphate, and silicate were quantified by colorimetry using an autoanalyzer.

### Macromolecular Analyses

Carbohydrate was analyzed colorimetrically using the TPTZ (2,4,6-Tris(2-pyridyl) –s triazine) method originally developed for dissolved carbohydrates (Myklestad et al., 1997), following a two-stage acid hydrolysis (Pakulski and Benner, 1992) and neutralization with sodium hydroxide.

Lipids were extracted and purified using modifications of the methods of Folch et al. (1957) to ensure full extraction (see Supplementary Text for detail) using only pre-combusted glass materials and HPLC-grade solvents. Dried lipid extracts containing 20–140 μg of total lipid were quantified by acid-dichromate colorimetry (Pande et al., 1963) using a spectrophotometer (Shimadzu UV-1800) with glyceryl tripalmitate (Sigma-Aldrich, Cat. #T5888) used as a reference standard. For analysis of the phosphorus contained in lipids, a portion of each total lipid extract described above was air-dried at room temperature to remove all chloroform and then extracted and analyzed as with total P samples.

Pigments were quantified by high performance liquid chromatography (HPLC) (Van Heukelem et al., 1994; Van Heukelem and Thomas, 2001) on an Agilent 1100 HPLC (Agilent Technologies, Santa Clara, CA, United States) using authenticated standards (DHI Lab, Horshølm, Denmark).

Protein was extracted from samples on polycarbonate filters by bead milling (Lysing Matrix D, MP Biomedicals) in 2% SDS (sodium dodecyl sulfate) buffer. Bead milling was performed four times for 1 min at 6.5 m s^-1^, with samples placed on ice for 2 min between each round of bead milling to prevent degradation by heating. Extracted protein was then quantified with the BioRad DC Assay, which is based on the Lowry method (Lowry et al., 1951), using a microplate reader (SpectraMax M3, Molecular Devices) and bovine gamma globulin (BioRad) as a standard. Protein quantification by these methods showed less than 5% variation compared to quantification after extraction with a more comprehensive protein solubilization buffer containing protease inhibitors (Brown et al., 2008) in preliminary tests with *T. pseudonana* and *Micromonas* sp., indicating that the simple SDS buffer used provides full protein extraction.

Nucleic acids were measured according to Berdalet et al. (2005) with modification of the sample extraction and scaled to a microplate format (see Supplementary Text for detail). RNA was quantified against an *E. coli* ribosomal RNA standard (Ambion #7940) and DNA was quantified against a type IX calf thymus DNA standard (Sigma # D4522). RNA was also quantified in samples from mid-exponential and mid-stationary growth phases after extraction with Trizol (Thermo Fisher Scientific), which is based on the phenol-chloroform extraction procedure (Chomczynski and Sacchi, 1987), to provide an additional verification of cellular RNA content. The extraction protocol provided by the Trizol manufacturers was used with modifications added to reduce the loss of RNA during various isolation and cleaning steps and account for consistent losses of RNA during solvent partitioning using a parallel RNA standard (Ambion #4940) spike-recovery test with each extraction (see Supplementary Information). Both RNA quantification methods provided similar results for all species at both growth conditions with the exception of N replete *O. tauri* and *Micromonas* sp., for which the Berdalet et al. (2005) method provided significantly higher (Student’s *T*-test, *p* < 0.01) RNA values (Supplementary Figure 1). Only results produced with the Berdalet et al. (2005) method are shown due to its greater consistency, simultaneous quantification of DNA, and greater apparent extraction efficiency (see Supplementary Information).

DNA quantification met or exceeded the expected genomic DNA content estimated from the known genome sizes of *T. pseudonana* (Armbrust et al., 2004), *O. tauri* (Derelle et al., 2002), and *Micromonas pusilla* (Worden et al., 2009), a species closely related to *Micromonas* sp., and assuming a mass of 10^-3^ pg for each mega-base pair of DNA (Doležel et al., 2003). A full genome sequence for *T. weissflogii* CCMP 1010 has not been published. *T. pseudonana* was assumed to be diplontic (Chepurnov et al., 2004) while *O. tauri* and *Micromonas* sp. were assumed to be haplontic (Graham et al., 2008). Measured DNA contents in N replete *O. tauri* and *Micromonas* sp. were 48.0 and 15.6% higher than genomic DNA estimates. Measured DNA content in *T. pseudonana* was 110% higher than its estimated genomic DNA content assuming diploidy. This higher DNA content in *T. pseudonana* may be the result of polyploidy, which has been observed in diatoms, particularly of the genus *Thalassiosira* (Von Dassow et al., 2008; Koester et al., 2010). The presence of multinucleate cells, which have been shown to occur in diatoms under nutrient or toxicity stress (Badour, 1968; Oey and Schnepf, 1970) or spontaneously (Von Dassow et al., 2006, 2008) may also explain *T. pseudonana* DNA content exceeding estimates of genomic DNA content.

The elemental content of macromolecular pools was calculated using a mean elemental stoichiometry for protein, carbohydrates, lipids, and nucleic acids (Geider and LaRoche, 2002) and the known elemental stoichiometry of phytoplankton pigments (Wright et al., 2005) as approximated in Figure 1. The cellular P not accounted for in RNA, DNA, and lipid P measurements was assumed to be residual P. This residual P likely represents intracellular storage of P as orthophosphate (PO_4_) or polyphosphate ([PO_4_]_2_[PO_3_]_n_) or the surface adsorption of orthophosphate (Sañudo-Wilhelmy et al., 2004; Dyhrman, 2016). As such, residual P was assumed to have the molecular formula of orthophosphate (PO_4_) in order to calculate its mass for comparison to the mass of other macromolecules.

### Bacterial Enumeration

The abundance of contaminating bacteria at all sampling points was measured with a flow cytometer (BD Accuri C6) according to Marie et al. (2005) using DNA fluorescence (produced after staining with SYBR Green I dye) and a lack of chlorophyll fluorescence to discriminate heterotrophic bacteria from microalgal cells. Assuming contaminating bacteria are coccoid with a relatively large mean cell diameter for marine bacteria (0.7 μm*)*, these bacteria would have a C content of 65 fg C cell^-1^ (Romanova and Sazhin, 2010). Using this estimate of C content, bacterial biomass was estimated to be 0.89–6.74% of particulate biomass (retained on a GF/F filter) in all samples. Additionally, the pore sizes of the filters (∼0.7 μm for glass fiber and 0.8 μm for polycarbonate filters) used to sample diatom cultures, which contained the highest bacterial abundances, are similar to or greater than the relatively large assumed bacterial cell size and thus unlikely to efficiently retain these bacteria. Given these measurements and assumptions, bacterial biomass was considered to be relatively low with no significant effect on the reported elemental or macromolecular measurements at each of these sampling points. Late-stationary sampling points for *T. pseudonana* and *O. tauri* were excluded from this study since bacterial biomass was deemed too high (10–13% of sampled biomass) to assume negligible effect on measurements of microalgal cell composition at these points.

### Data Analysis

Linear regressions as well as analysis of variance (ANOVA) and pairwise *post hoc* tests comparing proportional changes in elemental and macromolecular content among species were all performed using R statistical software (R Studio version 1.0.143). The error reported for all values indicates one standard deviation.

## Results

### Elemental Content and Stoichiometry

**FIGURE 3:**
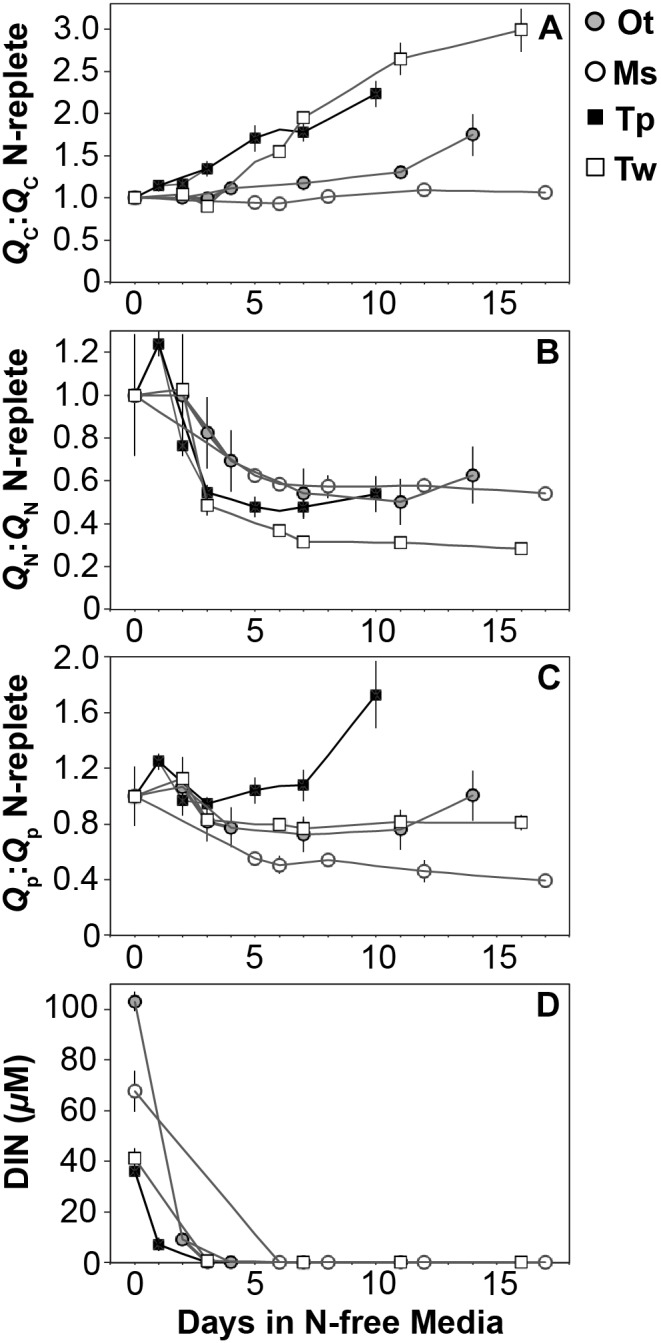
The ratio (mol:mol) of cellular **(A)** carbon, **(B)** nitrogen, and **(C)** phosphorus quota to N-replete quotas as well as **(D)** the available dissolved inorganic nitrogen from N-replete growth to N starvation. The decline in N quota to consistent minima in each species **(B)** and the removal of available DIN **(D)** indicate N starvation in all cultures. Error bars indicate one standard deviation among triplicate cultures.

The elemental and macromolecular content of the species in this study were observed from N-replete steady-state growth to non-steady state N starvation. All species showed a decline in N quota (*Q*_N_) to a consistent minimum (Figure 3B) as dissolved inorganic N fell below detection limits (Figure 3D) and replete levels of dissolved inorganic P (12.6 ± 4 μM) and silica (10.8 ± 2 μM) were maintained.

**FIGURE 4:**
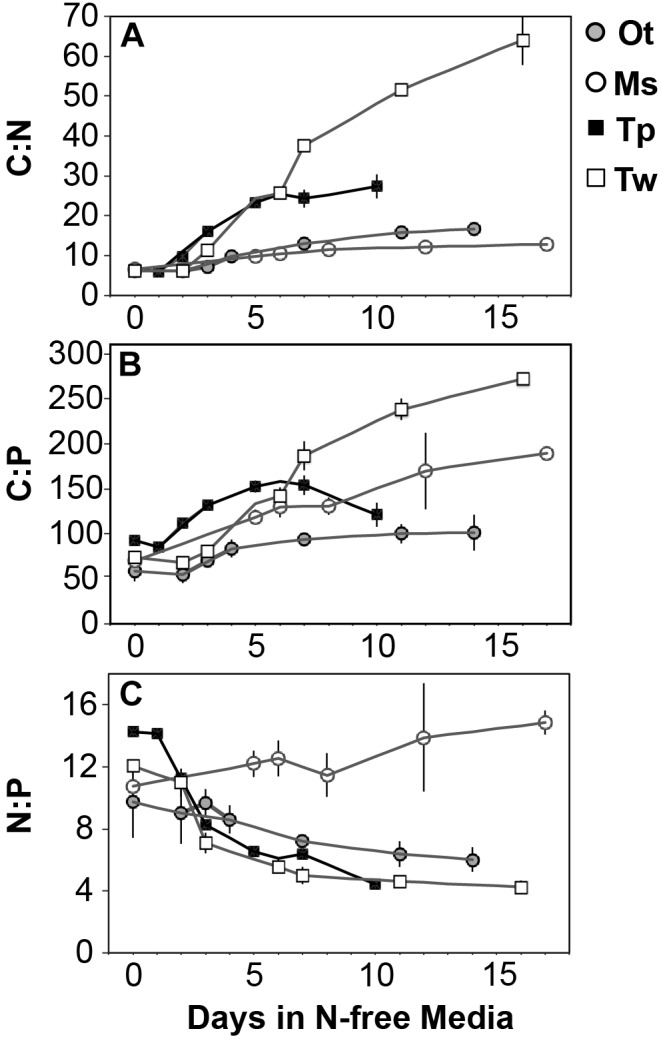
The change in molar ratios of **(A)** cellular carbon:nitrogen, **(B)** cellular carbon:phosphorus, **(C)** cellular nitrogen:phosphorus from N-replete growth to N starvation. Error bars indicate one standard deviation among triplicate cultures.

All species showed an increase in cellular C quota (*Q*_C_*)* with N starvation, but the extent of this increase varied considerably (Figure 3A and Table 1). Carbon accumulation with N starvation was greater in diatoms, particularly in the larger diatom species *T. weissflogii* (Table 2). The prasinophytes displayed far less change in *Q*_C_ than diatoms, particularly *Micromonas* sp., which had an increase in *Q*_C_ of only 8.95 ± 0.7% (Table 2). The decline in *Q*_N_ from N-replete growth to N starvation (Figure 3B and Table 1) was similar among *T. pseudonana* and both prasinophytes (-52.1 to -42.3%, Table 2), but significantly greater (*p* < 0.01) in *T. weissflogii* (-68.8 ± 5%). Though steady-state N-replete C:N was comparable across all species (6.32 ± 0.3, Figure 4A), under N starvation the greater C accumulation in diatoms resulted in a far greater increase in C:N compared to prasinophytes (Table 2 and Figure 4A).

**Table 1:** The cellular elemental content (in pg cell^-1^) and molar elemental ratios of each species during N-replete, steady-state growth and during N-starved stationary phase (mid-stationary sampling point, 6 days after cessation of growth).

	*O. tauri*		*Micromonas* sp.		*T. pseudonana*		*T. weissflogii*	
				
	N Replete	N Starved	N Replete	N Starved	N Replete	N Starved	N Replete	N Starved
Carbon	0.277	0.362	0.582	0.634	15.1	26.7	139	368
	(0.01)	(0.02)	(0.03)	(0.03)	(1)	(1)	(2)	(26)
Nitrogen	0.0536	0.0269	0.105	0.0604	2.70	1.29	26.6	8.30
	(0.011)	(0.002)	(0.004)	(0.003)	(0.1)	(0.2)	(1)	(0.5)
Phosphorus	0.0124	0.0094	0.0215	0.0099	0.418	0.450	4.89	3.98
	(0.002)	(0.001)	(0.001)	(0.0017)	(0.01)	(0.05)	(0.07)	(0.11)
C:N	6.17	15.7	6.49	12.2	6.51	24.3	6.10	51.6
	(1.0)	(0.46)	(0.10)	(0.013)	(0.21)	(2.2)	(0.21)	(0.93)
C:P	58.9	100	69.8	170	93.0	154	73.3	238
	(12)	(11)	(4)	(43)	(6)	(11)	(2)	(12)
N:P	9.71	6.39	10.8	13.9	14.3	6.4	12.0	4.61
	(2.3)	(0.8)	(1)	(4)	(0.3)	(0.3)	(0.33)	(0.14)


*Values in parentheses indicate one standard deviation.*

**Table 2:** Percent change in molar elemental content from N-replete mid-exponential growth (point ME in Figure 2) to N starved mid-stationary phase (point MS in Figure 2).

	*O. tauri*	*Micromonas* sp.	*T. pseudonana*	*T. weissflogii*
Carbon	30.7^a^	8.95^b^	77.5^c^	164^d^
	(2)	(0.7)	(5)	(11)
Nitrogen	-49.9^a^	-42.3^a^	-52.1^a^	-68.8^b^
	(10)	(3)	(6)	(5)
Phosphorus	-24.2^a^	-54.0^b^	7.6^c^	-18.7^a^
	(5)	(10)	(1)	(1)
C:N	155^a^	88.8^b^	273^c^	747^d^
	(26)	(1)	(27)	(29)
C:P	70.2^a^	144^b^	65.6^a^	225^c^
	(16)	(37)	(6)	(12)
N:P	-34.2^a^	29.1^a^	-55.5^b^	-61.7^b^
	(9)	(7)	(3)	(3)


*Values in parentheses indicate one standard deviation. Values with different superscript letters are significantly different (p < 0.01).*

Changes in cellular P quota (*Q*_P_) and P stoichiometry in response to N starvation were complex and species specific (Figure 2C and Table 1). In N-replete steady-state growth, prasinophytes were enriched in phosphorus resulting in a lower C:P and N:P compared to diatoms (Figure 4 and Table 1). With the onset of N starvation, *Micromonas* sp. showed the largest reduction in *Q*_P_ while the larger diatom *T. weissflogii* and the smaller prasinophyte *O. tauri* displayed smaller declines. In contrast, *Q*_P_ increased slightly (7.6 ± 1%) in *T. pseudonana* with N starvation (Table 2). The smaller changes in diatom *Q*_P_ resulted in a larger decline in N:P in diatoms as compared to *O. tauri*, while N:P increased slightly in *Micromonas* sp. as its *Q*_N_ and *Q*_P_ both declined greatly with N starvation (Figure 3 and Figure 4C). Despite these differing responses in *Q*_P_, C:P increased in all species with N starvation, although C:P declined at mid-stationary phase in *T. pseudonana* due to a large increase in *Q*_P_ at that sampling point (Figure 4B). As with C:N, C accumulation dominated the increase in C:P in all species except *Micromonas* sp., in which the increase in C:P was driven more by its relatively large decline in *Q*_P_ and little change in *Q*_C_ (Figure 4B and Figure 3A,C).

### Macromolecular Content

The sum of the calculated C and N content of macromolecules closely matched direct measurements of *Q*_C_ and *Q*_N_ (Supplementary Figure 2) such that macromolecular measurements accounted for 98.3 ± 8% of measured cellular C (Supplementary Table 1). Measurements of protein, RNA, DNA, and pigments accounted for 94.4 ± 8% of N in all samples except at N-replete mid-exponential phase for the diatoms *T. pseudonana* (86.5 ± 3%) and *T. weissflogii* (70.3 ± 3%). The unmeasured residual N in N-replete diatoms is likely inorganic N stored in central vacuoles (Miyata et al., 1986; Grover, 1991), free amino acids, or other N-containing small metabolites that can represent a substantial pool of intracellular N (Dortch et al., 1984; Granum et al., 2002; Lourenço et al., 2004). Measurements of RNA, DNA, and phospholipids accounted for only 29.2 ± 3% of cellular P (Supplementary Table 1). We define the cellular P that is not accounted for by these macromolecules as residual P. Considering our additional verification of our RNA and DNA measurements (see Supplementary Information for more detail), the direct measurement of phosphorus in total lipid extracts, and the expectation that the contribution of other organic phosphorus pools (e.g., ATP, other free nucleotides) will be small (Geider and LaRoche, 2002; Dyhrman, 2016) we assume that the residual cellular P is in the form of either intracellular storage (e.g., polyphosphate bodies, orthophosphate in vacuoles) (Miyata et al., 1986; Dyhrman, 2016) or as surface-adsorbed phosphate (Sañudo-Wilhelmy et al., 2004; Fu et al., 2005).

**FIGURE 5:**
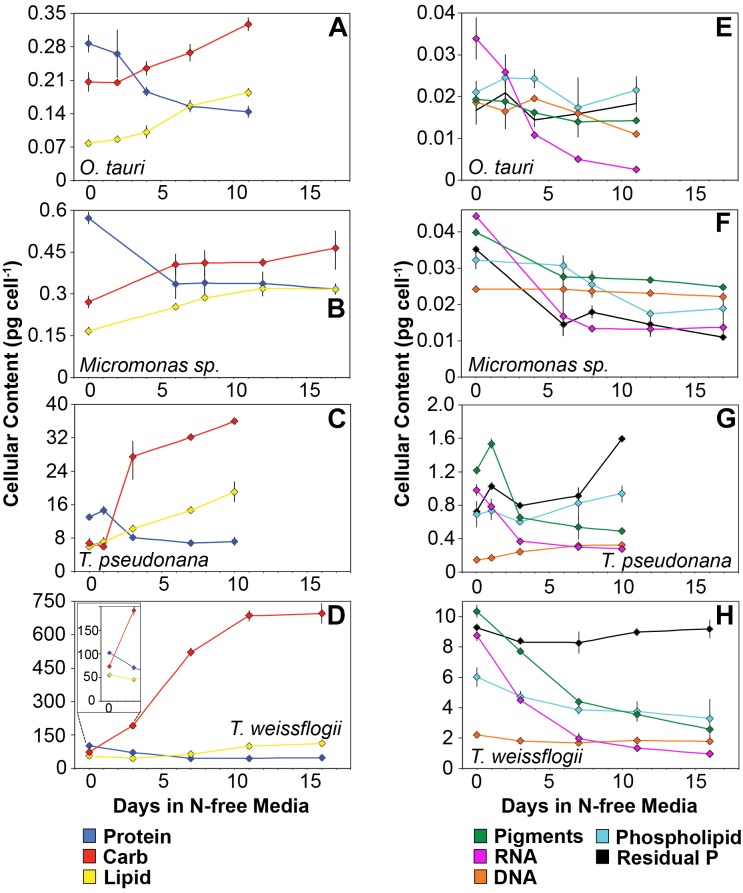
Changes in cellular content (pg cell^-1^) of **(A–D)** the major macromolecules protein (blue), carbohydrate (red), and lipid (yellow) as well as **(E–H)** the minor macromolecules pigments (green), RNA (purple), DNA (orange), phospholipid (cyan), and residual phosphorus (black) with N starvation. The inset in **(B)** highlights the macromolecular content of *T. weissflogii* at the beginning of the experiment as this is obscured by the large range of macromolecular content in this species. Error bars indicate one standard deviation among triplicate cultures. Mid-exponential and mid-stationary values for all macromolecular pools are also shown in Supplementary Table 2.

The major macromolecular pools of protein, carbohydrate, and lipid represented 94.7 ± 9% of *Q*_C_ and their variation matches the observed changes in *Q*_C_ and *Q*_N_ with N starvation (Figure 5 and Supplementary Table 2). Diatoms displayed far greater increases in carbohydrate quota (370–829%) than prasinophytes (52.2–58.4%) during N starvation (Figure 5 and Table 3). The relative changes in lipid quotas were more similar across species (112–202%). The greater increase of *Q*_C_ and C:N observed in diatoms during N starvation (Figure 3A, 4A) was due primarily to the greater carbohydrate accumulation in diatoms while lipid accumulation dominated the more modest increases in *Q*_C_ and C:N in prasinophytes (Figure 5 and Table 3). Protein accounted for 84.7 ± 8% of *Q*_N_ and its similar relative decline in all species with N starvation (Figure 5A–D and Table 3) mirrored the changes in *Q*_N_ as both parameters were highly correlated in all species (*r* = 0.90–0.98, *p* < 0.001).

**Table 3:** Percent change in macromolecular content from N-replete mid-exponential growth (point ME in Figure 2) to N starved mid-stationary phase (point MS in Figure 2).

	*O. tauri*	*Micromonas* sp.	*T. pseudonana*	*T. weissflogii*
Protein	-49.8^a^	-41.1^a^	-47.7^a^	-55.6^a^
	(5)	(6)	(6)	(5)
Carbohydrate	58.4^a^	52.2^a^	370^b^	829^c^
	(6)	(5)	(69)	(55)
Lipids	188^a^	132^b^	202^a^	112^b^
	(19)	(19)	(22)	(8)
Pigments	-25.7^a^	-32.9^a^	-56^b^	-65.6^b^
	(4)	(2)	(15)	(7)
RNA	-92.4^a^	-70.3^a^	-69.7^a^	-84.6^a^
	(20)	(8)	(11)	(3)
DNA	-41.2^a^	-4.76^b^	123^c^	-17.4^d^
	(3)	(0.2)	(14)	(1)
Phospholipid	2.24^a^	-46^b^	19^c^	-37.6^b^
	(0.4)	(4)	(6)	(8)
Residual P	9.66^a^	-58.9^b^	25.2^c^	-2.98^d^
	(2.3)	(14)	(3)	(0.1)


*Values in parentheses indicate one standard deviation. Values with different superscript letters are significantly different (p < 0.01). No percent change values for protein or RNA content were significantly different across species*

Minor pools of macromolecules (with respect to cell mass) made relatively small contributions to *Q*_C_ and *Q*_N_, but dominated species-specific dynamics in *Q*_P._ The small portion of *Q*_N_ not in proteins was mostly in nucleic acids and pigments. RNA was the most dynamic macromolecular pool in this study, showing a large and similar decline (-92.4 to -69.7%) in all species with N starvation. Pigments, particularly chlorophylls, also showed a large decline with N starvation (Figure 5E–H and Table 3) that was distinctly greater in diatoms (-65.6 to -56.0%) compared to prasinophytes (-32.9 to -25.7%). While these changes had a small impact on *Q*_N_, RNA and residual P content had a large influence on cellular P stoichiometry. The greater decline in N:P with N starvation in diatoms reflects their maintenance of larger residual P pools relative to prasinophytes, but similar declines in protein and N content across species. Declines in both RNA and residual P accounted for a large decline in *Q*_P_ and slight increase in N:P in *Micromonas* sp., an elemental response that differed from that of all other species. This decrease in both RNA and residual P in *Micromonas* sp. also caused an increase in C:P despite little C accumulation. The comparatively small change in residual P in *T. weissflogii* and *O. tauri* correspond to their small declines in *Q*_P_. The dynamics of residual P also dominated the response of *Q*_P_ in *T. pseudonana*, where a large increase in residual P during mid-stationary phase appeared to cause an increase in *Q*_P_ and a decline in C:P following its initial increase. Phospholipids and DNA represented the smallest portions of cellular P and were less variable than all other major macromolecules in response to N starvation (Figure 5E–H and Table 3) except in the case of *T. pseudonana*, in which the cellular DNA quota increased with N starvation while other species displayed only small declines.

### Elemental Allocation to Macromolecules

**FIGURE 6:**
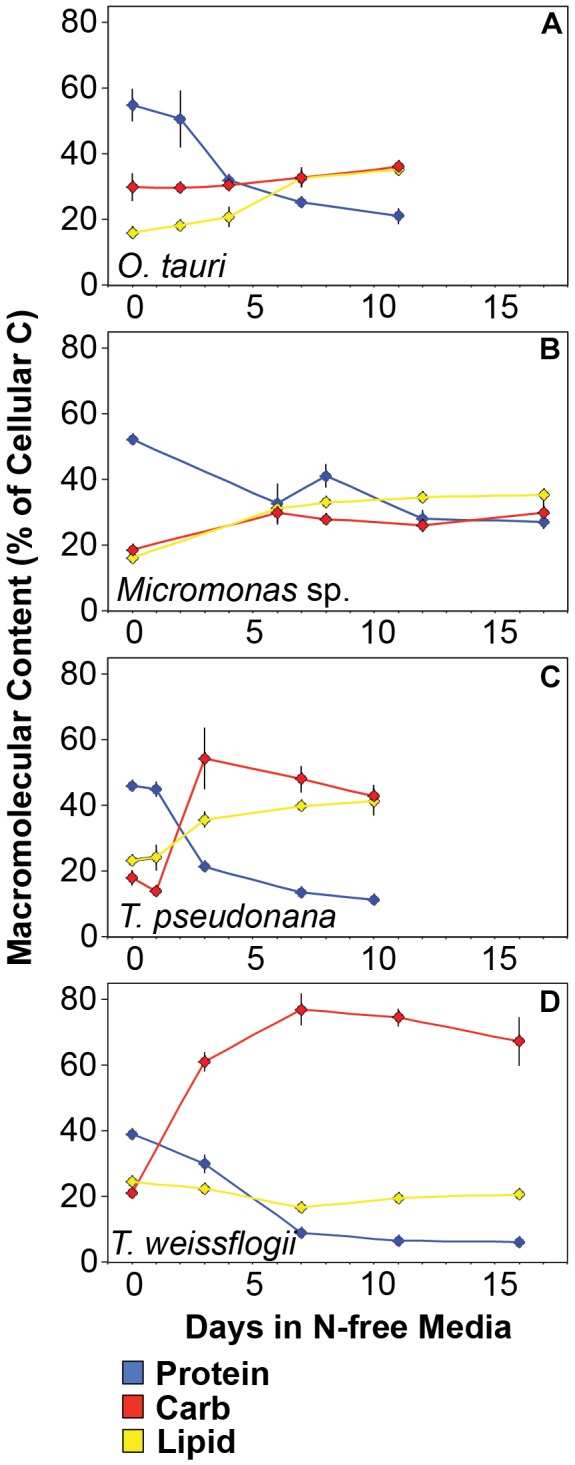
Changes in the allocation (%) of total cellular carbon among protein (blue), carbohydrates (red), and lipid (yellow) in **(A)** *T. pseudonana*, **(B)** *T. weissflogii*, **(C)** *O. tauri*, and **(D)** *Micromonas* sp. The estimated C content of the macromolecules shown accounted for 94.7 ± 9% of total cellular carbon. Error bars indicate one standard deviation among triplicate cultures.

**FIGURE 7:**
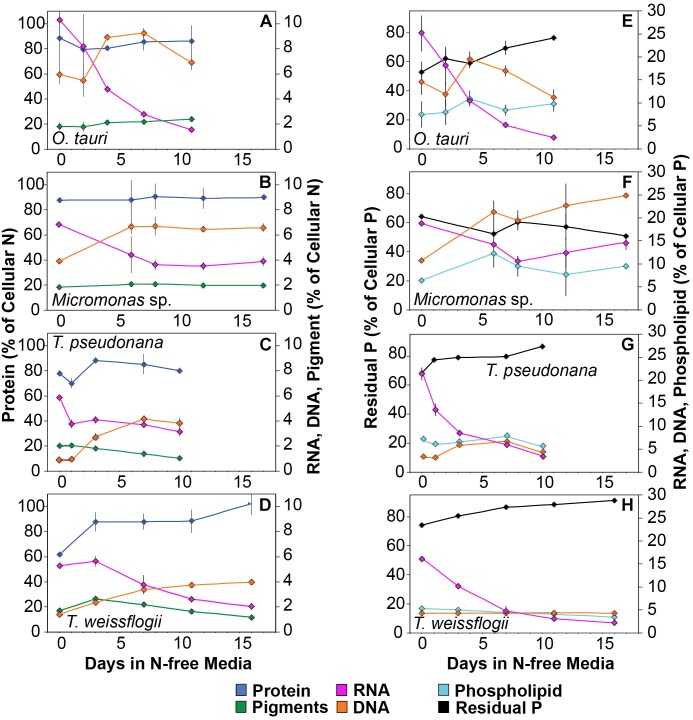
Changes in the allocation (%) of **(A–D)** total cellular nitrogen and **(E–H)** phosphorus among protein (blue), pigments (green), RNA (purple), DNA (orange), phospholipid (cyan), and residual phosphorus (black) with N starvation. Note that in the N allocation plots **(A–D)**, protein (blue) is plotted on a different scale on the left axis while RNA (purple), DNA (orange), and pigments (green) are plotted on a smaller scale on the right axis. Note that in the P allocation plots **(E–H)** Residual phosphorus (black) is on the left axis with a different scale than RNA (purple), DNA (orange), and phospholipids (cyan), which are plotted on a smaller scale on the right axis. Error bars indicate one standard deviation among triplicate cultures.

**FIGURE 8:**
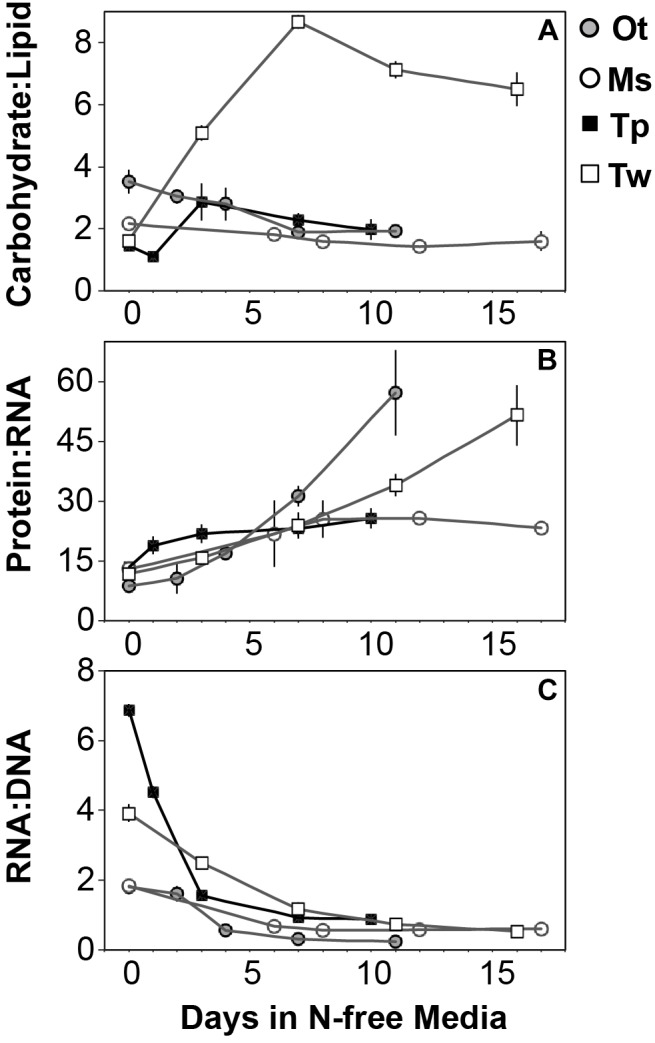
The mass ratios of **(A)** carbohydrate:lipid, **(B)** protein:RNA, and **(C)** RNA:DNA in each species with N starvation. Error bars indicate one standard deviation among triplicate cultures.

All species in this study shared similar general patterns in C and N reallocation in response to N starvation. Protein represented the largest pool of cellular C and N in all species under N-replete steady-state growth. With the onset of N starvation, C was reallocated from protein to lipid and carbohydrate (Figure 6) while a consistently high proportion of N was maintained in protein and a smaller proportion on N was reallocated from RNA to DNA (Figure 7A–D). Within these shared general patterns, far more C was allocated to carbohydrate in *T. weissflogii* (Figure 6D) while in the other species the proportion of *Q*_C_ in both carbohydrate and lipid increased in a similar fashion (Figure 6A–C). The hyper-accumulation and higher C allocation of carbohydrate in *T. weissflogii* is also apparent in its large increase in carbohydrate:lipid with N starvation (Figure 8A). In contrast, there was only a slight increase in carbohydrate:lipid in *T. pseudonana* and a slight decline in carbohydrate:lipid for both prasinophytes (Figure 8A). The similar general patterns in N allocation pattern are also reflected in the protein:RNA ratio of each species, which was similar among diatoms and *Micromonas* sp. (12.70 ± 0.8) and lower in *O. tauri* (8.64 ± 1.7) during N-replete growth and increased in all species with N starvation as RNA declined more rapidly than protein (Figure 8B). The overall N allocation pattern is also clear in the sharp decline of RNA:DNA in each species from steady state N-replete values of 5.39 ± 2.0 in diatoms and 1.82 ± 0.1 in prasinophytes to, similarly, low levels in all species (0.60 ± 0.3) with N starvation (Figure 8C). One notable difference in N allocation among species was the slight increase in pigments as a proportion of *Q*_N_ in prasinophytes due to their small change in pigment quotas with N starvation (Figure 7A,B), whereas pigments decreased as a proportion of *Q*_N_ in diatoms (Figure 7C,D).

The majority of *Q*_P_, in all species and at all growth phases, was allocated to residual P and this allocation was greater in diatoms (81.1 ± 7%) than in prasinophytes (60.5 ± 8%). The general pattern of changes in the allocation of *Q*_P_ during N starvation was similar among both diatoms and *O. tauri* as all showed a small increase in allocation to residual P, a declining allocation to RNA, and little to no change in the proportion of *Q*_P_ in DNA or phospholipids (Figure 7E–H). This general pattern was also reflected in the sharp decline in RNA:DNA observed in all species with N starvation (Figure 8C). *Micromonas* sp. showed a distinct pattern in P allocation during N starvation, with little change in allocation to RNA and residual P and an increase in the allocation to DNA (Figure 7F). The large declines in both residual P and RNA during N starvation in *Micromonas* sp. paralleled the decline in *Q*_P_, thus Residual P and RNA accounted for similar allocations of declining *Q*_P_ throughout N starvation. Since DNA quotas did not change in *Micromonas* sp., DNA made up an increasing proportion of the declining *Q*_P_ with the onset of N starvation (Figure 6F).

## Discussion

The diatoms and prasinophytes we examined represent distinct phyla, cell sizes and environmental niches, yet share some key similarities in the macromolecular responses to N starvation that dominate their elemental stoichiometry. The accumulation of C-rich carbohydrate and lipid with N starvation had the greatest impact on elemental stoichiometry, particularly C:N, in all species. However, diatoms displayed far greater increases in C content due mainly to carbohydrate accumulation while prasinophytes had more modest increases in C that were dominated by lipid accumulation. Unlike the distinct carbohydrate and lipid dynamics between diatoms and prasinophytes, the decline in protein and N quota overall was proportionally similar across species. There was also a similar relative decline in RNA in all species with N starvation. Notably, this similar relative decline in RNA exceeded the decline in protein in all species despite protein representing the vast majority of cellular N. The loss of RNA with N starvation did not dominate P dynamics as the majority of cellular P in all species was in residual pools, which are likely intracellular storage or surface adsorbed phosphate (Sañudo-Wilhelmy et al., 2004; Dyhrman, 2016). These residual P pools had strong, species-specific effects on cellular C:P and N:P, though the accumulation of C-rich macromolecules still dominated C:P, which increased in all species. Despite the general similarity of these biochemical responses across species, factors such as taxa and cell size clearly modify the extent of macromolecular responses and their influence on phytoplankton elemental stoichiometry.

### Carbohydrate and Lipid Accumulation Dominate Variability in C:N

The greater increase in C:N (273–747%) in diatoms were driven by carbohydrate accumulation and the smaller increases in C:N (88.8–155%) in prasinophytes were driven by lipid accumulation. Increases in carbohydrate content in both diatoms and prasinophytes are generally attributed to the accumulation of storage polymers (Myklestad, 1974; Ral et al., 2004). The larger intracellular space of diatoms may allow more carbohydrate storage accumulation (Sicko-Goad et al., 1984). Such accumulation may be restricted in the comparatively small prasinophytes (Raven, 1984; Ral et al., 2004), with *O. tauri* being the smallest known free-living eukaryote (Chrétiennot-Dinet et al., 1995). Carbohydrate storage in diatoms occurs in a cytosolic vacuole that can greatly expand to fill intracellular space (Chiovitti et al., 2004) while prasinophytes store carbohydrates in a highly localized sheath around the pyrenoid of their single chloroplast (Deschamps et al., 2008), a structural constraint that may restrict their ability to accumulate carbohydrates. Compared to carbohydrate stores, lipid bodies represent C storage that is more densely packed (Subramanian et al., 2013) as well as more C and energy-rich (Sorguven and Ozilgen, 2013). These properties make lipid bodies a more space-efficient form of C storage (Subramanian et al., 2013), thus smaller cell size and structural constraints may also explain the greater increase in lipid content (132–188%, Figure 5 and Table 3) than carbohydrate (52.2–58.4%, Figure 5 and Table 3) in the N-starved prasinophytes. The greater carbohydrate accumulation in diatoms during N starvation may also be due to their high exudation of dissolved carbohydrates compared to other phytoplankton taxa (Myklestad et al., 1989; Granum et al., 2002), which is enhanced by nutrient starvation (Biddanda and Benner, 1997; Penna et al., 1999). These dissolved carbohydrates can form extracellular polymeric substances (EPS) that can aggregate and account for a large fraction of particulate C and carbohydrates in diatom cultures and field populations (Passow, 2000: Engel, 2004; Wetz and Wheeler, 2007; Taucher et al., 2015).

The differing accumulation of C-rich macromolecules by diatoms and prasinophytes during N-starvation could also arise from distinct growth and energy utilization strategies. Carbohydrate storage may be preferred in the nutritionally dynamic niches where diatoms typically thrive (Sarthou et al., 2005) since carbohydrates are more rapidly accumulated and utilized than lipid stores (Siaut et al., 2011; Lacour et al., 2012a). Nitrogen starvation involves the down–regulation of photosynthetic components (e.g., proteins, pigments) to decrease cellular N demand and potentially limit harmful excess light absorption (Cullen et al., 1992). This decline in the photosynthetic apparatus during N starvation also involves the disassembly of lipids in photosynthetic thylakoid membranes and their reallocation to storage bodies prior to the *de novo* synthesis of storage lipids (Juergens et al., 2015). *Micromonas* sp. and *O. tauri* display far less decline in photosynthetic pigments and proteins (and thus presumably less decline in thylakoid membrane lipids) than diatoms during N starvation, instead relying on large inductions of non-photochemical quenching to dissipate excess light absorption (Liefer et al., 2018). The greater allocation of C to lipids in prasinophytes may be due to this strategy of maintaining photosynthetic components during N starvation. A smaller decline in thylakoid lipids prior to *de novo* synthesis of storage lipids may contribute to the greater allocation of C to lipids in prasinophytes during N starvation.

### Similar Relative Declines in Protein and RNA Constrain Variability in Cellular N

The relative decline in cellular N (*Q*_N_) and protein content was similar among all species in this study and thus had little effect on the differing responses in C:N between diatoms and prasinophytes. The similar percent decline in protein content across species (-41.1 to -55.6%) may indicate that the minimum protein quota for cell viability as a proportion of optimal protein quota is a shared physiological constraint on *Q*_N_ (Bonachela et al., 2013). We hypothesized that the decline in protein would exceed the decline in RNA during N starvation since protein represents the largest portion of *Q*_N_ and thus a metabolic response of minimizing *Q*_N_ seemed likely to disproportionately affect protein content. Instead, the similar decline in RNA across species (-69.7 to -92.4%) exceeded the decline in protein, which may be due to ribosome content (the largest cellular pool of RNA) being more closely tied to growth than any particular external resource (Elser et al., 2003).

The greater decline in RNA compared to protein observed here may also reflect a general resource allocation strategy in response to N stress. The redistribution and acquisition of N (protein-intensive processes) may be favored over maintaining biosynthesis and growth potential (ribosomes-intensive processes), as suggested by transcriptomic and proteomic studies of microalgae (Mock et al., 2008; Allen et al., 2011; Hockin et al., 2012). Biosynthetic and growth potential are directly related to ribosome/RNA content (Sterner and Elser, 2002), thus reducing RNA content during N starvation is an opportunity cost that may limit rapid recovery and growth when N is resupplied. This cost may be offset by N in RNA being reallocated to N metabolism and uptake during N starvation (Mock et al., 2008; Allen et al., 2011; Hockin et al., 2012), which may allow resistance and survival during N stress. Although the reduction in ribosome content was similar across species, the potential shared opportunity costs of this decline may be less severe for diatoms considering their adaptations for nutrient uptake. The success of diatoms in variable nutrient conditions has been attributed to their more rapid uptake (Lomas and Glibert, 2000) and greater storage capacity (Dortch et al., 1984; Lourenço et al., 2004) for N. These traits may lessen the opportunity cost of rebuilding N and P-rich ribosomes and explain the more rapid recovery of diatoms from N starvation as compared to prasinophytes (Liefer et al., 2018).

The small variation in N allocation across study species may be due to the effects of cell size and storage structures on intracellular N storage. During N-replete growth, N-rich macromolecules (protein, nucleic acids, chlorophylls) accounted for smaller proportions of *Q*_N_ in the diatoms *T. pseudonana* (86.5 ± 3%) and *T. weissflogii* (70.3 ± 3%) than in the prasinophytes (103 ± 11%) (Supplementary Table 1). We assume this greater residual N in diatoms represents intracellular storage of dissolved N, which appears to be greater in diatoms compared to other taxa (Dortch et al., 1984; Lourenço et al., 2004) due to their larger cell size (Grover, 1991; Tozzi et al., 2004) and use of a central storage vacuole (Miyata et al., 1986; Grover, 1991). The utilization of this greater intracellular N storage pool during N starvation may explain the significantly greater percent decline in *Q*_N_ in the larger diatom *T. weissflogii* (-68.8 ± 5%) compared to the other study species (-42.3 to -52.1%) (Figure 3B and Table 2). The size-dependent ability of diatoms to store dissolved N may allow greater declines in *Q*_N_ with N starvation as these N storage pools may represent a larger proportion of their N-replete *Q*_N_ (Supplementary Table 1).

### Residual P Dominates the Variability in C:P and N:P During N Starvation

Nitrogen starvation caused responses in C:P and N:P that were species-specific and controlled by uncharacterized residual P pools. Here we show that this residual P can account for the majority of phytoplankton *Q*_P_ (70.7 ± 13%), rather than the macromolecular pools of RNA, DNA and phospholipids (Figure 7E–H). We assume this residual P represents the intracellular storage or surface adsorption of inorganic phosphorus since these are the largest known pools of cellular P other than nucleic acids and phospholipids (Sañudo-Wilhelmy et al., 2004; Dyhrman, 2016). Luxury uptake and storage of P by phytoplankton has long been recognized from culture studies (Rhee, 1978; Goldman et al., 1979; Elrifi and Turpin, 1985) and more recently supported by field studies (Orchard et al., 2010; Martin et al., 2014; Diaz et al., 2016), but methodological challenges have made quantification of internal P stores like polyphosphates (polyP) elusive (Dyhrman, 2016). Cell surface-adsorbed inorganic P is another potential source of residual P that has been shown to account for a majority of *Q*_P_ in some cases (Sañudo-Wilhelmy et al., 2004; Fu et al., 2005).

Residual P also had differing effects on elemental stoichiometry among species with implications for the variability in phytoplankton C:N:P observed across growth conditions, taxa, and ocean biomes. The decline in residual P with N starvation in *Micromonas* sp. caused a slight increase in N:P. This response differed from the decline in N:P that was observed in the other study species in which residual P was stable or increased (Figure 4C, 5E–H). Changes in residual P with N starvation also resulted in more species-specific variation in C:P as compared to the responses of C:N (Figure 4A,B). Similarly, large contributions from residual P pools such as intracellular storage may contribute to the large variations in C:P and N:P observed by others among phytoplankton species (Quigg et al., 2003; Garcia et al., 2018), and even among strains of the same taxa (Martiny et al., 2016). Studies of steady-state N and P limitation have shown large and variable amounts of residual P (Rhee, 1978) or surplus P accumulation (Goldman et al., 1979; Elrifi and Turpin, 1985) and linked these processes to large variations in C:P and N:P within species. Assuming a large and variable amount of phytoplankton P storage has also allowed recent biogeochemical models to reproduce global C:N:P patterns as a function of phytoplankton physiological responses (Daines et al., 2014; Moreno et al., 2018). Our quantifications of functional P pools and residual P can help constrain such models and provide insights as to how P allocation may vary among key phytoplankton taxa.

A greater role of residual P rather than RNA in determining *Q*_P_ has important implications for relating phytoplankton elemental content and growth dynamics. The Growth Rate Hypothesis (GRH) (Sterner and Elser, 2002) anticipates a positive relationship between *Q*_P_ and intrinsic growth rate since *Q*_P_ is expected to mostly reflect the ribosomal RNA content needed for cell biosynthesis. However, the luxury uptake and storage of P during non P-limiting conditions can cause P content to be decoupled from growth and RNA content (Elser et al., 2000; Gillooly et al., 2005; Daines et al., 2014). This decoupling has been observed in microalgae during N and light limitation when residual P is maintained while growth and RNA content decline (Rhee, 1978; Goldman et al., 1979; Mouginot et al., 2015). Similarly, we find that *Q*_P_ is decoupled from growth during the onset of N starvation as most of *Q*_P_ is allocated to residual P pools with varying dynamics among species. Considering this, the GRH prediction of a positive relationship between growth and *Q*_P_ may not apply to marine phytoplankton or rather this prediction is less applicable to N-limited communities than P-limited conditions for which *Q*_P,_ RNA content, and growth may be more tightly coupled. Better characterizations of P accumulation and storage allocation are needed to understand the true P growth requirements of phytoplankton (Gillooly et al., 2005; Cherif et al., 2017).

### The Large Potential Impacts of C Accumulation and Residual P on Ocean Biogeochemistry

The differing resource allocation strategies we observed among diatoms and prasinophytes may greatly impact their respective roles in export production and other ocean biogeochemical processes. The greater carbohydrate accumulation in diatoms during N starvation could cause their exported biomass to have a higher C:N than that of prasinophytes. A higher C:N in sinking diatom biomass would result in lower C use efficiency for its consumers and a greater export of C to the deep ocean (Hessen et al., 2004; Finkel et al., 2010). The greater export production of diatoms compared to other phytoplankton taxa (Michaels and Silver, 1988; Buesseler, 1998) is often attributed to their larger mineralized cells and tendency to form aggregates, which result in faster sinking rates (Thornton, 2002; Sarthou et al., 2005). Greater carbohydrate production with N stress may be a physiological mechanism in diatoms that contributes to this rapid sinking. Intracellular accumulation of carbohydrates provides negative buoyancy (Raven, 1984; Richardson and Cullen, 1995) and carbohydrate exudation creates the EPS that allows fast-sinking aggregates to form (Passow, 2000). Recent work indicates that smaller taxa like prasinophytes make larger contributions to export production than previously thought due to their inclusion in such aggregates (Turner, 2015; Richardson, 2019). Unlike diatoms, the sinking and export of prasinophytes seems less likely to be enhanced by N stress given their preference for lipid rather carbohydrate accumulation observed here.

Our findings provide taxonomic and physiological constraints on phytoplankton C:N:P that can strengthen efforts to mechanistically quantify and predict ocean biogeochemical processes. The similar proportional decline in protein across species in this study may represent a useful constraint for predicting variable phytoplankton C:N and N:P in cellular trait-based models. In contrast, the presence of large residual P pools with species-specific variation presents a significant challenge for integrating phytoplankton biochemical traits into predictive models (Follows and Dutkiewicz, 2011). Global patterns in particulate C:P have been linked to increasing phytoplankton P requirements for biosynthesis (i.e., ribosomes) at low temperature (Toseland et al., 2013) and regional variation in P availability for phytoplankton growth (Galbraith and Martiny, 2015). Accounting for both the biochemical effects of temperature adaptation and variation in P storage with P availability has allowed recent modeling efforts to capture the full variability and much of the global patterns in phytoplankton and particulate C:P (Moreno et al., 2018). Our findings show that taxonomic variation in C accumulation and residual P pools like P storage can also greatly affect phytoplankton C:P and thus C export efficiency even in the absence of these proposed mechanisms. This highlights the impact that taxonomic variation in biochemical traits and the biogeography of phytoplankton communities may have on ocean C:N:P patterns (Weber and Deutsch, 2010; Martiny et al., 2013a). The presence of large, variable C and P storage pools provide additional mechanisms that may cause the observed flexibility in ocean particulate C:P (Galbraith and Martiny, 2015). Our work shows that these mechanisms may be affected by phytoplankton community structure and N availability in unexpected ways.

## Author Contributions

JL, AI, MJF, and ZF contributed to the conceptualization and experimental design of the study. JL, AG, MHF, IB, and CB contributed to sample collection. JL, AG, and MHF performed all biochemical analyses. JL wrote the first manuscript draft. AI, MJF, AO, and ZF contributed to the writing and revisions. All authors read and approved the submitted version.

## Conflict of Interest Statement

The authors declare that the research was conducted in the absence of any commercial or financial relationships that could be construed as a potential conflict of interest.
