# Supplementary figures and images for: The Macromolecular Basis of Phytoplankton C:N:P Under Nitrogen Starvation
Source: Front Microbiol. 2019 Apr 17;10:763. doi: 10.3389/fmicb.2019.00763 (PMC6479212; doi:10.3389/fmicb.2019.00763)

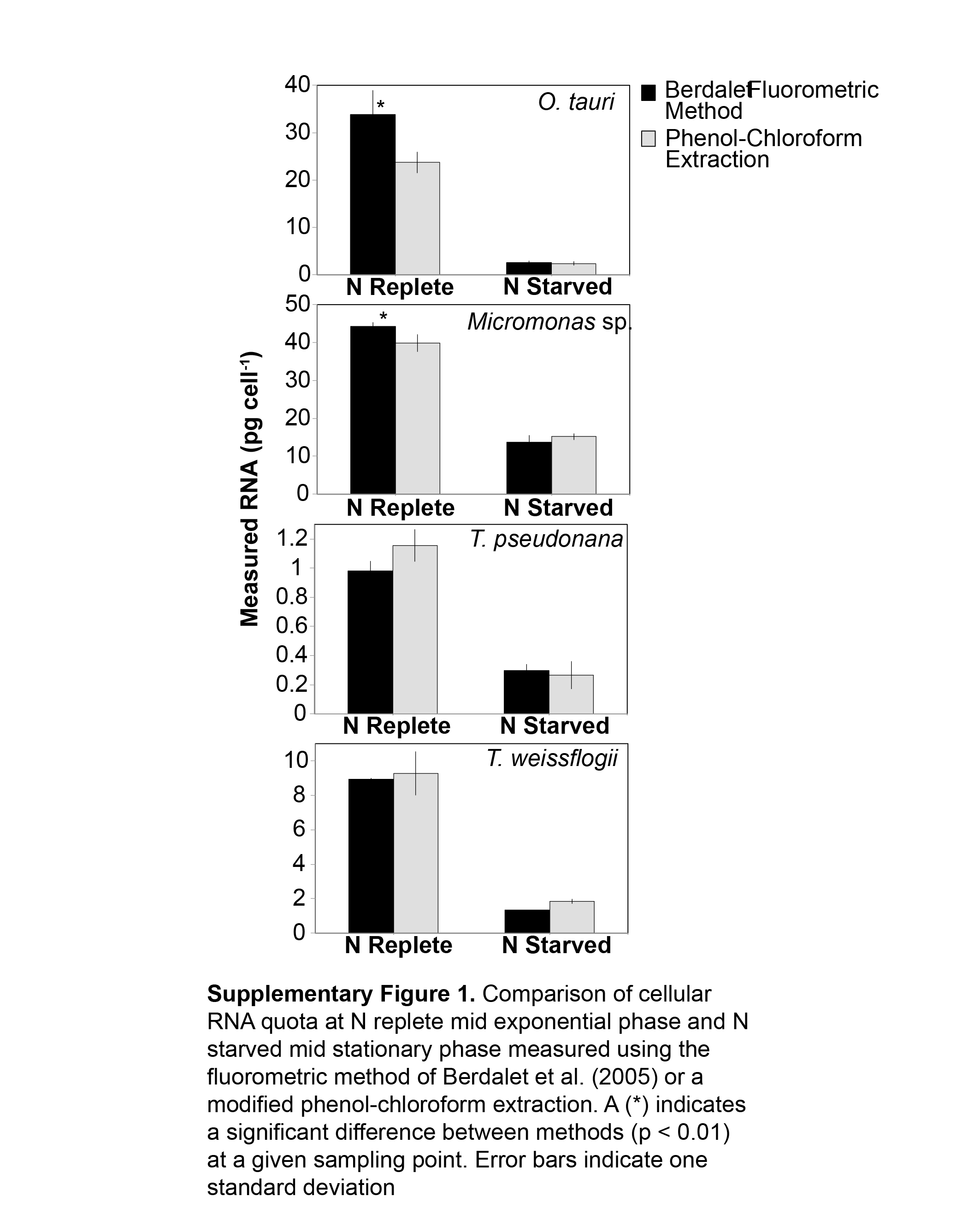

Supplement: Supplementary file 1 [file Image_1.JPEG]

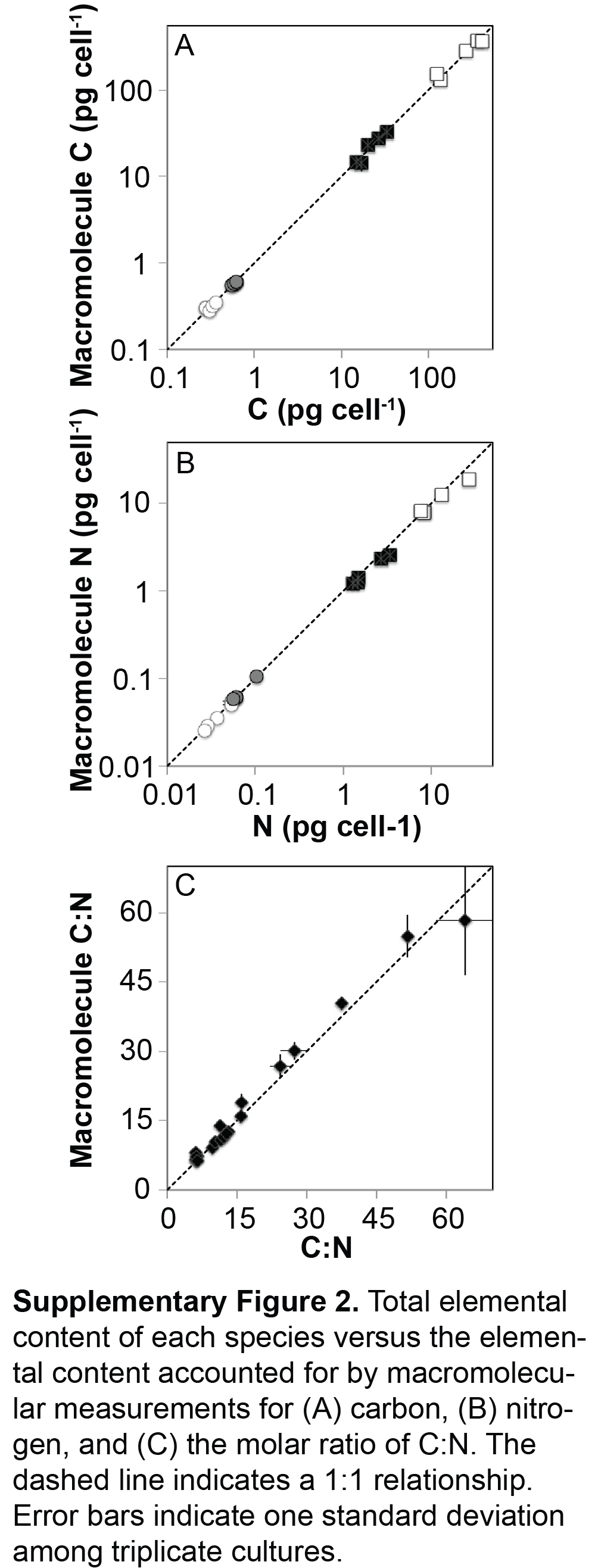

Supplement: Supplementary file 2 [file Image_2.JPEG]
