# Supplementary material for: The Macromolecular Basis of Phytoplankton C:N:P Under Nitrogen Starvation
Source: Front Microbiol. 2019 Apr 17;10:763. doi: 10.3389/fmicb.2019.00763 (PMC6479212; doi:10.3389/fmicb.2019.00763)
Supplement: Supplementary file 3 [file Table_1.DOCX]

Supplementary Material

**The macromolecular basis of phytoplankton C:N:P under nitrogen starvation**

**Justin D. Liefer^1*^, Aneri Garg^1^, Matthew H. Fyfe^1^, Andrew J. Irwin^2^, Ina Benner^1^, Christopher M. Brown^1^, Michael J. Follows^3^, Anne Willem Omta^3^, Zoe V. Finkel^1^**

^1^Departments of Geography and Environment, Mount Allison University, Sackville, NB, Canada

^2^Departments of Mathematics and Computer Science, Mount Allison University, Sackville, NB, Canada

^3^Department of Earth, Atmospheric and Planetary Science, Massachusetts Institute of Technology, Cambridge, MA, United States

**^*^Correspondence:**

Justin D. Liefer

jliefer@mta.ca

**Modifications to Lipid Methods**

The lipid extraction protocol used was that of Folch et al. (1957) modified to include the addition of ultra-pure water to freeze-dried samples before the addition Folch extraction solvent (2:1 chloroform:methanol) to generate the expected ratios of chloroform:methanol:water in the final extraction solvent since the Folch et al. (1957) method assumes the sample is wet tissue. After the addition of water, samples were rapidly frozen and re-thawed twice before addition of solvents to enhance extraction (Ryckebosch et al. 2012). Additionally, extractions were performed four times and included 2 minutes of sonication using a sonic bath to further enhance extraction. Pooled extracts were purified by partitioning with 0.88% KCl (Folch et al. 1957), dried under a continuous stream of N_2_ gas, and redissolved in 100% chloroform to provide additional purification.

**Description of RNA/DNA Methods**

The method of Berdalet et al. (2005) used in this study involves extraction of RNA and DNA using a detergent-based extraction buffer. Sub-samples of each extract were diluted, treated with RNase, DNase, or RNase+DNase, and stained with SYBR Green II. RNA and DNA were then determined by SYBR Green II fluorescence, with corrections applied based on the background fluorescence present after nuclease treatment. Sample filters were extracted by bead milling (Lysing Matrix D, MP Biomedicals) in the final extraction buffer (0.5% n-lauryl sarcosine, 5mM Tris, 1mM EDTA) four times for 30 seconds at 6.5 m s^-1^, with samples placed on ice for 2 minutes between each round of bead milling. Samples were then kept at room temperature for 50 minutes during which they were vortexed at high speed for a duration 30 seconds every 10 minutes. The assay was scaled to a microplate format and measurement of RNA and DNA fluorescence was performed in a 96-well opaque black microplate using a microplate reader (SpectraMax M3, Molecular Devices).

Additional RNA analyses were performed on N-replete mid-exponential and N-starved mid-stationary samples (see Figure 1) using Trizol (ThermoFisher Scientific) and a modified version of the manufacturer’s protocol to reduce the loss of RNA during various isolation and cleaning steps and account for consistent losses of RNA during solvent partitioning. Samples on polycarbonate filters were extracted in 1ml Trizol solution and immediate bead milling four times for 30 seconds at 6.5 m s^-1^, with samples placed on ice for 2 minutes between each round of bead milling. After partitioning of the Trizol extract with chloroform and centrifugation, ~75% of the RNA-containing upper aqueous phase of the extract was removed to avoid collection of the DNA-containing interphase. An aliquot of pure Trizol-chloroform aqueous upper phase was then added to remaining Trizol-chloroform extract. The pure Trizol-chloroform aqueous upper phase added to the extracts was produced earlier by making a 5:1 mixture of pure Trizol reagent and chloroform, allowing this to partition overnight, then removing and storing this pure upper aqueous phase at -20°C. The Trizol-chloroform extract was then mixed for 30 seconds by vortexing, centrifuged, and ~75% of the resulting RNA-containing upper aqueous phase was removed and pooled with previously removed upper phase. This partitioning process was repeated once more and served to remove more RNA from the upper aqueous phase while limiting removal of contaminating DNA from the interphase while also capturing more RNA that may have been lost to the phenol-chloroform phase during initial partitioning. The combined aqueous extracts were then partitioned again with chloroform to remove remaining phenol contamination. The RNA was then precipitated from aqueous extract with isopropanol, with 100 μg RNA-grade Glycogen added as a co-precipitant. RNA was allowed to precipitate for 48 hours at -20°C. The resulting RNA pellet received two washes with 75% ethanol to remove any residual phenol and salts. When redissolving the RNA pellet in RNase-free water, the additional steps of freezing the extract at -80°C after the addition of water and then warming to 60°C for 5 min were added to enhance RNA dissolution. In parallel with the sample RNA extractions and analyses, triplicate spiked blank samples containing clean polycarbonate filters and 7 μg RNA standard (*E. coli* ribosomal RNA, Ambion #7940) were also processed as described above. This was done to account for the consistent fraction of RNA lost during the various isolation and purification steps (~35% lost based on spike recovery) and the extraction efficiency of the spiked blank samples was used a correction factor for the sample RNA measurements. This modified phenol-chloroform extraction and the method of Berdalet et al. (2005) provided similar results for all species at both growth conditions with the exception of N replete *O. tauri* and *Micromonas* sp., for which the Berdalet et al. (2005) method provided significantly higher (Student’s T-test, p < 0.01) RNA values (Figure S1). Additional samples were processed only with the Berdalet et al. (2005) method due to its greater ease, consistency, apparent extraction efficiency, and simultaneous quantification of DNA.

**Supplementary Table 1.** The % of total cellular C, N, and P that were accounted for by all macromolecular measurements for each study species under both N-replete (mid-exponential) and N-starved (mid-stationary) conditions. Values in parentheses indicate one standard deviation. Elemental content of each macromolecule was based on their known or mean stoichiometry (Geider and LaRoche 2002).

**Supplemental Table 2.** Macromolecular content (in pg cell^-1^) of each species during N-replete, mid-exponential growth and during N-starved mid-stationary phase (5 days after cessation of growth). * - difference of measured total lipid content and total pigment content. ** - estimated from lipid phosphorus content assuming a mean phospholipid elemental stoichiometry (Geider and LaRoche 2002). *** - Phosphorus storage content estimated as the difference of total P content and P in Lipids, RNA, and DNA and assuming all phosphorus storage is present as polyphosphate. Values in parentheses indicate one standard deviation. Trends in macromolecular content for all sampling points are shown in Figure 5.
